# Ring Currents in the Clar Goblet Calculated Using Configurational State Averaging

**DOI:** 10.1021/acs.jpca.4c05393

**Published:** 2024-11-15

**Authors:** Timothy K. Dickens, Roger B. Mallion, Patrick W. Fowler, Barry T. Pickup, Joseph Mowll-Clarke

**Affiliations:** † Peterhouse, Cambridge CB2 1RD, U.K.; ‡ Department of Chemistry, University of Sheffield, Sheffield S3 7HF, U.K.

## Abstract

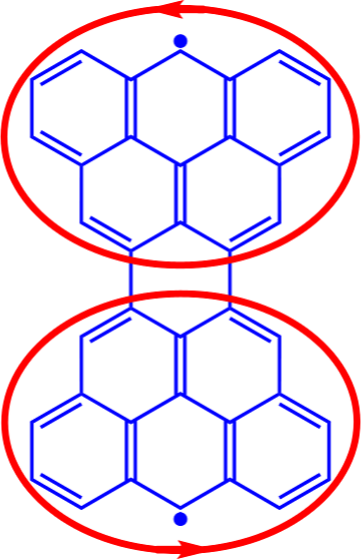

The closed-shell Hückel–London–Pople–McWeeny formalism for ring currents is extended to Aufbau configurations with open shells calculated as configurational averages. The method is applied to the non-Kekulean benzenoid known as the Clar goblet, recently synthesized on the Au(111) surface. Multiplicity of the ground state is a complication: for the Clar goblet, Hund’s rule of maximum multiplicity implies a triplet whereas Ovchinnikov’s rule implies a singlet. This disagreement has little effect on the predicted ring currents. Ring-current maps are calculated for the 36π dication, 40π dianion, and low-lying states of the 38π neutral, using Hückel–London and Hubbard–London models. All show twin diatropic perimeter currents on separate halves of the molecule. These are compared with ipsocentric pseudo-π and ab initio maps of induced π-current for closed-shell singlet configurations of dianion, dication, and neutral. Configurationally averaged Hückel–London calculations give a good account of the consistent diatropic ring currents in the Clar goblet for the three charge states.

## 1 Introduction

**Figure 1:**
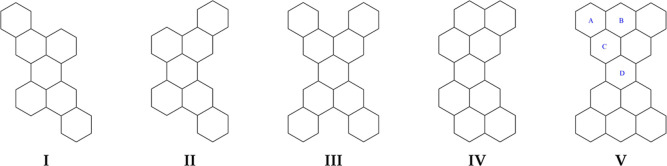
Carbon skeletons^25^ of five structures ostensibly related to perylene, whose ring currents were requested by Clar.

Synthesis by in situ modification of a precursor adsorbed on the Au(111) surface^1^ has given access to the elusive benzenoid known as the Clar goblet (**V** in Figure 1), creating an opportunity to compare theoretical predictions for this species in different spin states with experimental results, and reopening questions about the magnetic properties of non-Kekulean benzenoids.^2^

The Clar goblet is the smallest concealed non-Kekulean benzenoid.^3,4^ As we will show later, Ovchinnikov’s rule^2^ predicts a singlet ground state for every such benzenoid.^3,4^ Experiment supports this expectation, indicating that the ground-state of the Clar goblet is an open-shell singlet that lies 23 meV (0.0085β) below a triplet.^1^ This energy is comparable to the Landauer limit for single-bit erasure in computer memory.^5^ Triangulene is another molecule famously hypothesized by Clar and recently achieved by in situ surface synthesis.^6^ For triangulene, in contrast to the goblet, absence of a Kekulé structure is revealed by simple counting, and Ovchinnikov’s and Hund’s rules agree in predicting the high-spin (triplet) ground state.

Calculations on the electronic structure of the Clar goblet had been published before,^7^ but the new experiment has generated a flurry of theoretical studies using a variety of techniques ranging from empirical to high-level ab initio.^8−11^ Our approach here is to work with the simplest models to gain physical understanding of the magnetic properties of this intriguing species. We begin with the simplest one-electron model (Hückel theory) which requires nothing more than the adjacency matrix of the graph, and in its Hückel–London extension can be used to predict the current induced by an external magnetic field.^12^ Next, we take the simplest model of electron interaction, the Hubbard model.^13^ Ovchinnikov^2^ based his arguments on the Heisenberg–Dirac (HD) Hamiltonian:^14−16^ the Hubbard model^13^ interpolates between Hückel and HD limits as the Hubbard interaction parameter, *U*, goes from zero to infinity.^17^ In the Hückel limit, the π-electrons occupy delocalized orbitals that distribute electron density around the system. In contrast, in the HD limit, each site carries exactly one electron of up or down spin. All three models can be considered as purely graph-theoretical when they are applied to π systems of planar benzenoids under the assumption of regular hexagonal ring geometry.

Results from the Hückel–London model and its “Hubbard–London” extension will be interpreted here using the symmetry-based selection rules for current that were originally developed in ab initio ipsocentric^18,19^ approaches to aromaticity of closed-shell systems. We confront the predictions of the simple models with direct ipsocentric calculations, albeit confined to closed-shell type: the Hückel– and Hubbard–London current maps are compared to those deduced from the pseudo-π method,^20^ a proxy for full ab initio calculation, and maps from ab initio calculations themselves.

It is important to note that the orbital ring-currents relevant to aromaticity are associated with an interaction energy that is of second order in the magnetic field; in systems of high multiplicity such as triangulene, they will be masked by the first-order interaction energy of electron spins. In this sense, singlet Clar goblet is an ideal case for investigation of aromaticity, which was a major interest of Clar.^21^ Before presenting our results, we give some historical context.

More than 50 years ago, one of the present authors (RBM), while at the Oxford University Mathematical Institute, had the privilege to be in frequent communication with Professor Erich Clar (1902–1987), the doyen of synthetic organic chemists in the domain of the condensed benzenoid hydrocarbons, who held a chair at the University of Glasgow. At the end of a long and distinguished career, Clar was on the verge of publishing his influential work, The Aromatic Sextet,^21^ which became his monument in the field.^22^ At the time, RBM was making a systematic experimental study of the ^1^H NMR spectra of condensed benzenoid structures,^23^ alongside with calculation of their theoretical ring-current properties by application of McWeeny’s extension^24^ of the Hückel–London approach^24^ to the magnetic properties of conjugated systems. This approach was later formalized into the topological ring-current model, the method of calculation being given the acronym HLPM (Hückel–London–Pople–McWeeny).^25,26^

It was widely acknowledged that Clar could make benzenoid hydrocarbons that nobody else was capable of synthesizing, and he had built up a remarkable collection of samples. In 1971, RBM and Clar had an arrangement whereby they regularly exchanged ^1^H NMR spectra of rare benzenoids for calculations of their ring-current intensities and the contributions of these to calculated proton chemical shifts. In a letter dated 8 July 1971,^27^ Clar sent an apparently routine request for the ring-current intensities in a family of five structures, all ostensibly related to perylene (whose central rings exhibit fixed single bonds).^21,28,29^ These are **I** to **V** in Figure 1.

Calculations for structures **I** to **IV** were straightforward, but when an attempt was made to find ring-current intensities for structure **V** a difficulty arose: the frontier orbitals turned out to be a degenerate nonbonding pair, each of which would be singly occupied, if the standard Aufbau principle were used and Hund’s rule applied. Accordingly, the structure labeled as **V** in Figure 1 was predicted to be an open-shell species. At the time, the reason that the open shell of **V** created a problem when it came to calculating ring currents was that the expressions that arise in the McWeeny formalism^24^ were presented^24^ only for closed-shell systems. Clar was, therefore, informed^30^ that the ring currents for **V** were not available.

## 2 Methods

### 2.1 Graph Theory

The methods employed in this work span a range of mathematical and quantum chemical models. The first component of the mathematical toolkit is graph theoretical, specifically the spectral graph theory of benzenoids.

As pointed out by Dewar and Longuet–Higgins,^31^ species such as **V** cannot be drawn with a classical Kekulé structure. Radical and non-Kekulean character are related by the mathematical fact that, for all benzenoids, there is a simple link between Kekulé count (number of perfect matchings of the molecular graph) and the determinant of the adjacency matrix (essentially, the tail coefficient of the characteristic polynomial of the molecular graph).^32^ The Kekulé count is the square root of the tail coefficient taken without sign. Graph **V** has vanishing adjacency determinant, i.e., the matrix has some eigenvalues equal to zero, and is therefore singular. A benzenoid that has a singular molecular graph has no Kekulé structures, and vice versa.

A singular benzenoid also has a specific electronic structure. For bipartite graphs, the order of the graph (i.e., the number of carbon centers participating in the π system), *n*, and the nullity of the graph (the number of nonbonding π orbitals), η, have the same parity (by the Coulson-Rushbrooke Pairing Theorem^33^). From the combination of Aufbau and Pauli principles and Hund’s rule, in the absence of Jahn–Teller distortion, the ground-state of a neutral system with a bipartite graph, treated in the simple Hückel model, has the so-called natural configuration,^34^ with all bonding MOs doubly occupied and a nonbonding shell occupied by a number of electrons equal to the degeneracy of the nonbonding level (number of zero eigenvalues of the adjacency matrix). In general, the natural configuration may give rise to a number of states of various spin multiplicity, all with equal π energy within the Hückel model, but averaging over these states gives invariant results for charges, bond orders, bond numbers, polarizabilities and induced currents, within the Hückel and Hückel–London models. These averages are subject to caveats discussed below.

The Clar goblet is the first of the infinite class of concealed non-Kekulean benzenoids.^35^ Necessary conditions for existence of a Kekulé structure/perfect matching, i.e. even number of vertices of the molecular graph, and equal numbers of peaks and valleys in the canonical drawing of the graph,^35^ are satisfied by this graph, but in fact no such matching exists. By parity, all concealed non-Kekulean benzenoids have a degenerate nonbonding shell consisting of an even number of nonbonding orbitals (NBMOs). The number of NBMOs (η) is revealed by direct construction.^36,37^ or from vertex and edge independence numbers.^32^

A useful way to classify the NBMOs of a benzenoid comes from the definition of the color excess of a bipartite graph. In a bipartite graph, the vertices can be divided into two sets (say black and white) such that every edge of the graph has one vertex in each set. Then the color excess, Δ, is just the difference in size of the two sets. Let the adjacency matrix of graph *G* be **A**(*G*). With an appropriate ordering of vertices, **A**(*G*) for bipartite *G* has all its nonzero entries in one off-diagonal block (and its transpose). Consideration of the rank of this block shows that the number of zero eigenvalues of **A**(*G*) is at least Δ. Clearly, the color excess of a bipartite graph has the same parity as *n* and therefore η. Hence, the nullity of *G* is restricted to values η = Δ, Δ + 2, ···. The difference η – Δ is the count of supernumerary zeros^36^ of the adjacency matrix. (See also discussion in refs (38 and 39)).

The pairing theorem for bipartite graphs,^33^ implies that for each eigenvalue λ of **A**(*G*) for graph *G* there is an eigenvalue −λ, and that an eigenvector *x*′ corresponding to −λ can be produced by taking an eigenvector *x* corresponding to λ and reversing the signs of entries on one partite set. Hence, NBMOs (the kernel vectors) of the bipartite graph can be described in a basis consisting of Δ orbitals that are localized on the larger partite set of vertices, plus (η – Δ)/2 pairs of supernumerary vectors of which one is localized on the larger set, and one on the smaller. In such a basis, each kernel vector is self-paired under the pairing theorem.

By definition, every concealed non-Kekulean benzenoid has Δ = 0 but η ≠ 0, and all NBMOs are supernumerary. In the case of the Clar goblet, there are two supernumeraries, which can be represented in several ways: (i) in a basis where each has density on only one partite set, (ii) by symmetry-pure linear combinations, where each basis function belongs to an irreducible representation *D*_2*h*_, or (iii) as functions respectively localized on upper or lower “triangulene” motifs.

The existence of supernumerary zeros is significant in the theory of electronic structure of benzenoids, since Ovchinnikov’s rule,^2,40,41^ predicts that the spin multiplicity of the ground state will be 2*S* + 1 = Δ + 1. We can interpret this rule as saying that the multiplicity will be as predicted by Hund’s rule of maximum multiplicity, if supernumerary NBMOs are completely ignored. As the Clar goblet has only supernumerary zeros, Ovchinnikov’s rule implies a singlet ground state for it, and in fact for every concealed non-Kekulean benzenoid, rather than the state of higher multiplicity predicted by direct application of Hund’s rule. An open-shell singlet ground state for the Clar goblet is consistent with the experimental observations.^1^

### 2.2 Group Theory

The second component of the mathematical toolkit is group theoretical, and refers to the analysis of orbital symmetries and excitations that contribute to induced current density.

As it will be useful in the discussion of the calculated magnetic properties, a summary is given of symmetry aspects of the π molecular orbitals and energy levels of the goblet. Considered as a flat structure embedded in 3*D* space, the most symmetric realization of **V** has *D*_2*h*_ point-group symmetry. We take *x*, *y*, *z* as short in-plane, long in-plane and out-of-plane axes, respectively. Several versions of the character table of this group appear in the literature; we take the ordering of columns {*E*, *C*_2_(*z*), *C*_2_(*y*), *C*_2_(*x*), *i*, σ(*xy*), σ(*xz*), σ(*yz*)}.^42^ A minimal basis of πp_*z*_ functions spans the reducible representation
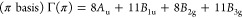
1

In Hückel theory of all-carbon frameworks, the π orbital energies are related to eigenvalues of the adjacency matrix, {λ_*k*_}, by ϵ_*k*_ = α + λ_*k*_β for *k* = 1, ...*n*, where α and β are coulomb and resonance integrals, respectively. This relation reduces to ϵ_*k*_ = −λ_*k*_ when α and β are taken as the zero and unit of energy. Hence, eigenspaces with positive, zero and negative eigenvalues correspond to bonding, nonbonding and antibonding orbitals, respectively. For the Clar goblet, the characteristic equation of the adjacency matrix factorizes as
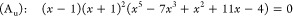
2
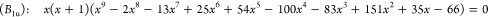
3
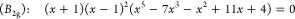
4
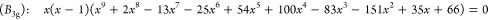
5from which the reducible representations of nominal bonding, nonbonding and antibonding subspaces are (by application of the Descartes rule of signs)
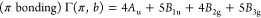
6
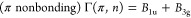
7
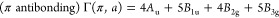
8

The Pairing theorem^33^ has an immediate group theoretical corollary. If the set of MOs with eigenvalue λ and representation Γ is paired with the set that has eigenvalue −λ and representation Γ′, then Γ′ = Γ_*_ × Γ, where Γ_*_ is the symmetry of the pairing operator, the 1-dimensional irreducible representation of a vector with entries +1 on all vertices in one partite set, and −1 on all vertices in the other.^43^ Γ and Γ′ may be reducible. For the Clar goblet in *D*_2*h*_ symmetry, Γ_*_ is ungerade and corresponds to the symmetry of a translation along the *y*-axis.

These reducible representations have implications derived from the ipsocentric selection rules for the sense of molecular-orbital contributions to π current. In the given setting of *D*_2*h*_, translations in the molecular plane span the reducible representation Γ(*x*) + Γ(*y*) = *B*_3u_ + *B*_2u_, and the rotation in the molecular plane has Γ(*R*_*z*_) = *B*_1g_. Hence, the selection rules for diatropic and paratropic current in terms of unordered pairs of occupied and empty orbitals are
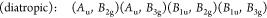
9
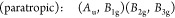
10Diatropic and paratropic excitations are mutually exclusive, by the centrosymmetry of *D*_2*h*_, but a given occupied orbital can give rise to contributions of both types, by excitation to different target virtual orbitals.

It follows from these rules that if the NBMO pair of the Clar goblet were split by Jahn–Teller interaction to give a closed-shell singlet, for example, any contributions from excitations within the HOMO – LUMO pair would be purely diatropic. In fact, there is no indication prima facie of distortion in the pristine Clar goblet. The two largest eigenvalues of the bond–bond polarizability matrix (expressed in units of β^–1^) are ≈0.9146 and 0.9141, which fall short of the threshold for distortion of ≈1.8 given by the standard model due to Binsch et al.^44^

### 2.3 Configurational State Averaging

The next component of the toolkit of methods is quantum chemical calculation of induced currents by the Hückel–London model. This employs the expedient of configurational state averaging, to allow for the fact that the ground state of the neutral Clar goblet arises from partial occupation of the nonbonding shell. The use of configuration state averages (CSA) goes back to the earliest numerical quantum chemical work on atoms by Hartree.^45^ The method circumvents the technical problems arising from the existence of open shells (states where a degenerate level is not fully occupied). Open-shell states give rise to the possibility of different electronic spin arrangements, some of which may have partially filled degenerate orbitals. Orbitals within a degenerate shell, however, are only defined up to a unitary transformation. The CSA approach takes an average over all possible electronic arrangements within an open shell, hence rendering the calculated properties invariant to any unitary transformation among the degenerate set of orbitals. The method of configurational state averages has been used more recently in ab initio quantum chemistry,^46^ taking into account two-electron interactions. Our interest here is restricted to Hückel theory and extensions that allow electron interactions via inclusion of a subset of two-electron integrals.

We define shell components in terms of eigenstates of the graph adjacency matrix. We distinguish three such shells. The closed shell, C, has its constituent molecular orbitals (MOs) doubly occupied. The open shell, O comprises a set of orbitals with single eigenvalue λ_O_, and degeneracy *g*_O_. The virtual shell, V, includes all unoccupied MOs, i.e. all MOs not in C or O. The respective shell MOs are defined so that their constituent eigenvalues satisfy λ_c_ > λ_o_ > λ_v_, for all c ∈ C, o ∈ O, and v ∈ V. The electronic configuration implied by these definitions is Aufbau, since the doubly occupied levels are lower in energy than the open shell orbitals, which are lower in energy than the empty ones. The assumption is that the electronic occupation, *n*_O_, of the open shell is greater than zero, but less than 2*g*_O_. The difficulty with partially filled open shells is that they imply a choice of which orbitals within O are occupied. Such a definition renders expectation values dependent on the exact choice of MOs inside the degenerate shell and these are only defined up to a unitary transformation. This difficulty is circumvented by using a configurational average, which is a mean value obtained by summing over all possible occupation schemes within shell O. The effect is to introduce an average occupation number, ν_O_, for each MO in the open shell
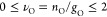
11and therefore to render expectation values unitarily invariant with respect to transformations within O. In general, ν_O_ may take fractional values.^46,47^ The natural configuration,  is an Aufbau configuration such that shell C comprises the entire set of positive-eigenvalue orbitals, and the shell O has a half occupied null space (λ_O_ = 0), with ν_O_ = 1. If the graph has no null space,  is a closed shell.

**Figure 2:**
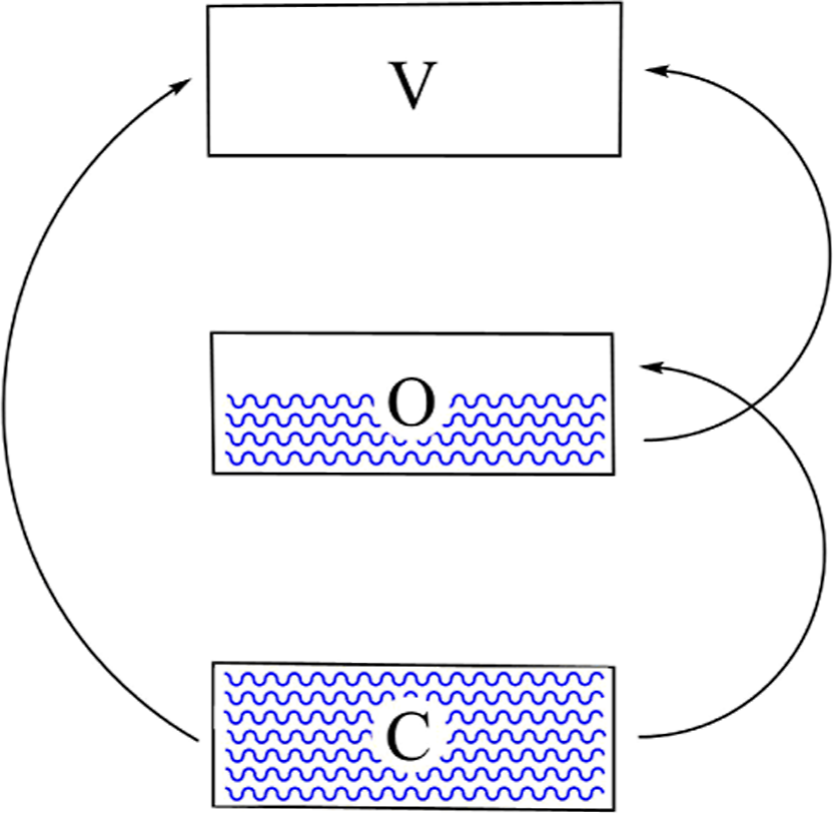
Particle-hole scheme for the virtual excitations in the summations of eq 12.

We can now set up the scheme of calculation for induced currents of general open-shell configurations by using the CSA approach. We adopt a convention that labels MO coefficients *c*_pk_ by vertex (p) and eigenvector (k). Here, MO coefficients can always be defined to be real, in which case it is convenient to adopt a simplified notation for the antisymmetrised product *c*_rs,jk_ = (*c*_rj_*c*_sk_ – *c*_sj_*c*_rk_). The imaginary bond–bond polarizability is then, in our notation (compare eq (3.15) of ref (24)):
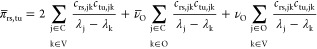
12where π̅ is indexed by pairs of vertices, and  is the average hole occupation of MOs in the open shell. Equation 12 is valid for both closed- and open-shell electron configurations. Figure 2 represents eq 12 in terms of virtual particle-hole excitations. Bond orders and imaginary bond–bond polarizabilities defined in this way can then be used to derive currents, as in the standard implementation of the HLPM approach.^25^ Step-by-step guides on how these calculations are carried out are available,^25,48^ and they detail the roles of bond order and imaginary bond–bond polarizability together with the strategy for choice of idealized geometries (in this case based on regular hexagons). The calculations produce ring currents, which are used to deduce bond currents by application of Kirchhoff conservation of current.

An equivalent strategy, used in the Sheffield programs, is to use finite-field calculations of bond currents from numerical diagonalization of the complex Hückel–London Hamiltonian for the molecule in the external field, which can be performed in real arithmetic,^12^ even though the Hamiltonian and MOs are intrinsically complex for nonzero values of the magnetic perturbation. This is achieved at the price of doubling all matrix dimensions. Defining equations for the bond currents are given, for example, in ref (49). The finite-field technique has been used to find bond currents in benzenoids,^50^ fullerenes,^12^ and for anapole response of toroidal frameworks.^49^ When fractional occupation is used with the finite-field approach, the results correspond to the configurational state average. Ring currents follow from the bond currents by a reverse application of Kirchhoff’s law.

### 2.4 Hubbard Model

The next component of the methods toolkit is designed to investigate the effect of two-electron interactions in lifting the degeneracy of the Hückel model. We chose a simple model that is again essentially graph theoretical. This is the Hubbard model, introduced in 1963 as a way of describing highly correlated states in solids with narrow bands.^13^ The Hubbard Hamiltonian comprises the Hückel Hamiltonian (essentially the adjacency matrix) with addition of the Hubbard potential
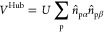
13which is defined in the AO basis associated with graph vertices, and the number operator
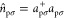
14counting electrons on vertex p with spin σ. The constant *U* is specified in units of the Hückel β (known in the physics context as the hopping integral, *t*), which is itself negative, and the physical effect of a negative *U* is therefore to introduce a repulsive two-electron potential that penalises electron pairs residing on vertices. In terms of ab initio quantum chemistry, this would amount to amounts to replacing the full collection of two-electron AO basis integrals, , by a single one-center integral *U* = ⟨pp|pp⟩ that is independent of vertex p.

Mishra et al. used the mean-field approximation^51^ to the Hubbard model (MFH), which replaces the two-electron operator in eq 13 with
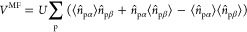
15where the angle brackets indicate the state average . This is an SCF-like potential that contains one-electron operators only, with expectation value
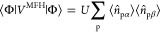
16

There are advantages to using the MFH Hamiltonian for systems with translational symmetry, since the expectation value, , is then identical in each cell of the infinite system. For finite systems, however, there is no reason not to use the full Hubbard Hamiltonian, and that is what we do here.

The standard Hubbard model can be extended to cover the case of molecules (molecular graphs) in external magnetic fields. This is effectively an enhancement of the Hückel–London model. This Hubbard–London model for current maps can be implemented within the finite-field approach, by combining the Hubbard SCF procedure with the Hückel–London treatment of the magnetic perturbation. The justification of the approach is that the magnetic field terms are one-electron, whereas the Hubbard potential is purely two-electron. It should be noted that, as with the Huckel–London MOs, Hubbard–London MOs become complex in the presence of the field.

### 2.5 Ipsocentric Current Maps

Finally, although exhaustive ab initio treatment of induced currents in the ground state of the Clar goblet is beyond the scope of the present work, some coupled Hartree–Fock calculations were performed for comparison with Hückel– and Hubbard–London models, and proved to be informative. Calculations were carried out at two levels. The pseudo-π model, using the standard σ-only basis,^20^ was used to calculate π-current maps.^52^ An idealized carbon framework based on regular hexagons of side 1.4 Å was used in these calculations. In the ab initio calculations,^52^ carried out in the 6-31G** basis, that framework was extended to include CH bonds of length 1.09 Å at 120° CCH bond angles.

## 3 Results and Discussion

**Figure 3:**
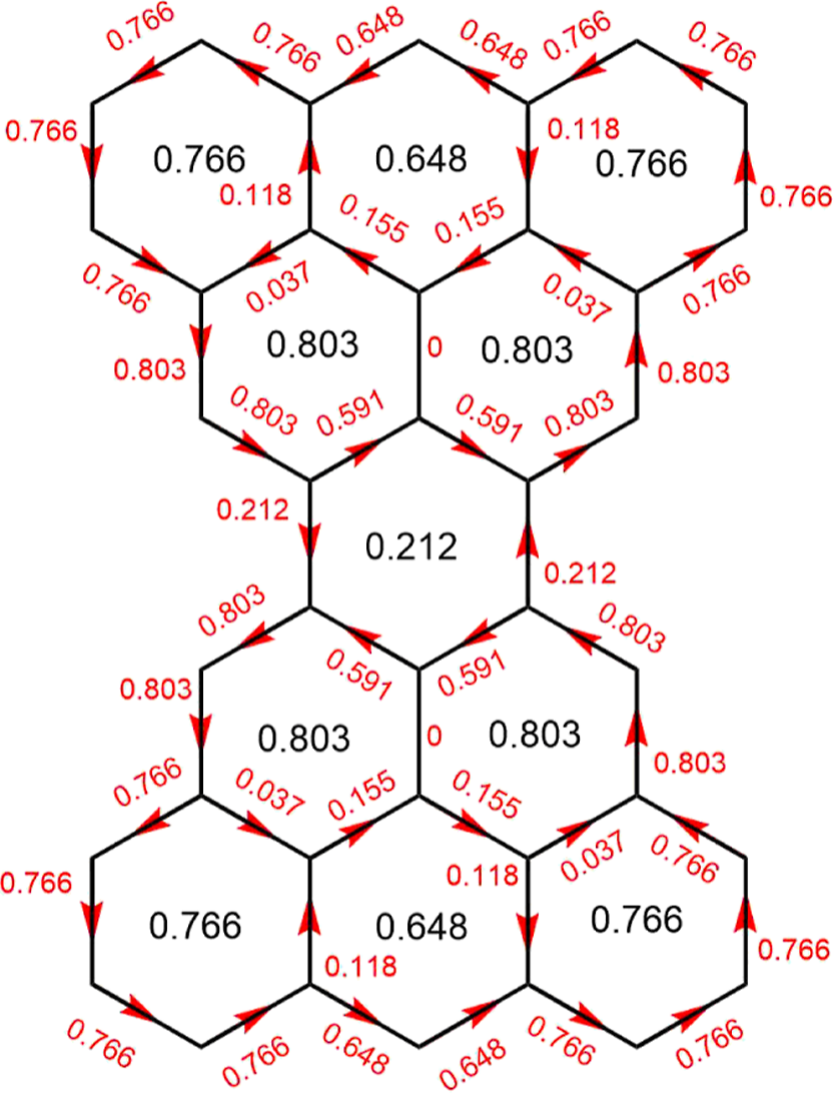
HLPM ring- and bond-current map for the neutral Clar goblet under configurational state averaging. Currents are expressed as ratios to those for benzene. Ring-current intensities are in black, and bond currents in red. This map is identical to those obtained for dicationic and dianionic Clar goblet within the same model (see text).

Ring-current intensities calculated by the Hückel–London method for the Clar goblet are displayed in Figure 3. The intensities of the individual ring currents expressed as a ratio to that calculated for benzene by the same method^25^ are written, in the center of the appropriate ring. As is to be expected for a condensed benzenoid hydrocarbon, all ring currents are positive (that is, diatropic, and in our convention anticlockwise). Also presented in Figure 3 are the currents that may be regarded as flowing along individual bonds, as if the carbon framework were a classical electrical network,^53^ with currents at junctions respecting Kirchhoff’s Current Conservation Law. In this classical analogy (black) ring currents play the role of classical loop currents^53^ while (red) bond-currents represent flow in the wires of the network.^53^ In the original computer code, ring currents are calculated first and bond currents are (uniquely) constrained to be Kirchhoff-consistent with them. Symmetry is not imposed but its emergence in the calculated maps gives an extra check on the calculations.

The overall pattern seen in Figure 3 consists of two islands of current, each diatropic and strong, located on the perimeter of a 5-hexagon fragment, with only minor leakage across the central isthmus.

Relevant to the discussion is a specific mathematical property of Hückel–London theory for bipartite graphs. It can be proved that the contribution to current from an electron in the nonbonding space of a bipartite system is exactly zero. This has been demonstrated for the cycle-based Aihara formulation of the Hückel–London model^54,55^ and is also easy to see for the present formulation in terms of bond–bond polarizabilities. Simply note that eq 12 for  is linear in ν_O_, and therefore for any graph the CSA interpolates linearly between the electron counts corresponding to empty and full O shells. For bipartite graphs, where the O shell is nonbonding, the pairing theorem^33^ implies that  is the same for ν_O_ = 0 and ν_O_ = 2, and hence there is no variation in predicted current with ν_O_. The chemical implication for the Clar goblet is clear: identical current maps are found in the Hückel–London model for dication, dianion and the CSA neutral molecule.

At this level then, an answer to Clar’s request for information on **V** could have been given using the closed-shell maps. This short-cut would not apply for nonalternant systems, such as fullerenes. Moreover, exact equivalence of dication and dianion maps is not retained in treatments that include explicit two-electron interactions. For example, the pseudo-π maps discussed below are not identical for dicationic and dianionic **V**, although they are similar, as are the full coupled Hartree–Fock ipsocentric maps, a relic in both cases of an exact property of the simpler model.

It should be noted here that occupied-orbital contributions to current are defined differently in Hückel–London and ipsocentric^19^ approaches: in Hückel–London theory the contribution of a given set of orbitals depends only on their occupation, and not on the occupation of any other set; in the ipsocentric approach, the contribution of a given occupied orbital or shell depends on the availability of empty orbitals of appropriate symmetry and relative energy. Thus, occupied nonbonding orbitals, even for a bipartite molecular graph, may give rise to significant current. For interpretation, it has been found useful to take the symmetries of frontier orbitals from Hückel theory and then apply ipsocentric selection rules based on symmetry products to rationalize current maps and the orbital contributions derived from a calculation at a higher level.^56^ If a π system is fully delocalized, this gives an account of currents in terms of frontier orbitals. For large π systems, this methodology extends to a band-contribution picture. In such cases, features such as the intense diatropic perimeter current of a carbon nanoflake are band-to-band and arise from a group of several canonical molecular orbitals, but the complex pattern of eddies in the flake interior is accounted for by just a few electrons at the top of the occupied band.^57^ As we shall see, perimeter patterns of this type are found in ipsocentric calculations for the Clar goblet.

Hückel–London maps for benzenoids cannot distinguish among different electron counts for the nonbonding space, nor, as the calculation of total properties is essentially spin-free, among the sheaf of spin states for the neutral. In this sense, the CSA is the most appropriate treatment when working within the Hückel–London model. However, experiments have been interpreted in terms of a specific open-shell singlet state for the Clar goblet.^1^ For this reason, we need to consider models that include the two-electron interactions that lead to a separation in energy of the spin states. The simplest of these is the Hubbard model, and its “Hubbard–London” extension to calculation of current as outlined in the methods section above.

We will consider low-lying states of the Clar goblet π-system in the context of the Hubbard model. It is possible to construct a core doubly occupied closed-shell state, |Φ_C_⟩, with 36 π-electrons for the dication
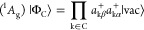
17where the operator *a*_k*σ*_^+^ creates an electron of spin σ in MO ψ_k_. There are several 38 π-electron states that can be constructed for the neutral system, bearing in mind that NBMOs 6*b*_3g_ and 6*b*_1u_ (where we are using the standard notation of lower-case letters for the irreps of MOs) span a doubly degenerate null space. With the abbreviations *a* = 6*b*_3g_, and *b* = 6*b*_1u_, we can define closed shell configuration state functions (CSF)
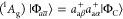

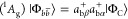
18

It is useful to define a further four 38 π-electron CSFs. They have a 36 π-electron core, and 2 π-electrons placed in the nonbonding shell in all possible ways. The states with *M*_S_ = 0 comprise a triplet and three singlets. CSFs for these can be written as
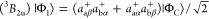

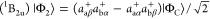

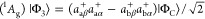

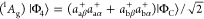
19

The state |Φ_1_⟩ is the *M*_S_ = 0 component of the open-shell triplet, whereas |Φ_2_⟩ is the open shell singlet. The symmetry of each CSF is indicated with its defining equation.

We note that the final two states in eq 19 are linear combinations of the simple closed-shell CSF defined in eq 18. However, in this definition, the states |Φ_1_⟩ and |Φ_4_⟩ are invariant to unitary transformations between the MOs *a* and *b*, whereas |Φ_2_⟩ and |Φ_3_⟩ mix. It follows that if we introduce two-electron interactions, and conduct SCF procedures on |Φ_1_⟩ and |Φ_4_⟩, the energy functionals for these states will each have three unitarily invariant shells of MOs: a doubly occupied core, a two-electron open shell comprising MOs *a* and *b*, and a virtual (empty) shell. The other two states, |Φ_2_⟩ and |Φ_3_⟩, will have four shells, the core–shell, shell *a*, shell *b*, and the virtual shell. The two states must lie on a single SCF optimization surface, but may exhibit more than one stationary solution, as we shall see.

We complete our list of states by defining a 40 π-electron closed-shell state for the Clar goblet dianion
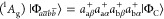
20

All CSFs defined in eqs 18–20 are degenerate at the Hückel level of approximation. In the Hubbard approximation, the diagonal matrix elements of the four CSFs defined in eq 19 are
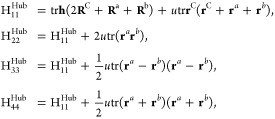
21where, allowing for the complex nature of the MO coefficients in the finite-field Hubbard-London calculations that we use later, the density matrices are
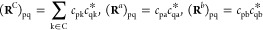
22and the remaining quantities are diagonal parts of density matrices
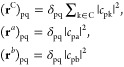
23which are all positive. The equation for the configurational state energy can be obtained from eq 21, recognizing the spin multiplicities of each state, as
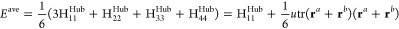
24

The full 4 × 4 CI matrix constructed using the CSF in eq 19 can be understood using the symmetry and spin designations. It comprises 1 × 1 blocks for |Φ_1_⟩ and |Φ_2_⟩, and a full 2 × 2 block for the ^1^*A*_g_ CSFs, |Φ_3_⟩ and |Φ_4_⟩. For the latter pair, we have a choice of performing SCF iterations for the separate states, or a full MCSCF procedure. The CSFs in eq 19 allow the use of multishell SCF theory as defined by McWeeny^58^ in Sheffield, where the effective Fock operator for this is known as the McWeenyan.^59^

Calculations used either symmetry-adapted linear combinations of AOs (SALCs), explicitly blocking the Fock matrices into the four blocks *A*_u_, *B*_1u_, *B*_2g_ and *B*_3g_, or in an AO basis without imposition of symmetry constraints. For consistency with the work of Mishra et al.,^1^ we used β = −2.7 eV, and *U* = 3.5 eV, corresponding to *U* = −1.3β. These workers carried out “spin polarized” calculations where different basis sets were adopted for α and β spin orbitals. In quantum chemistry such calculations would be termed unrestricted Hartree–Fock (UHF), and we adopt this notation in our table of results. All calculations denoted UHF involved single-determinant CSFs.

The mean field Hubbard (MFH) and full Hubbard formalisms are identical for single-determinant CSFs, but for Φ_1_, Φ_2_, Φ_3_ and Φ_4_ they give different results. Each of these four CSFs comprises two determinants, each a double excitation with respect to the other. Nonzero double-excitation matrix elements are obtained when a two-electron operator, such as *V*^Hub^, is used. The MFH potential, *V*^MFH^, does not fulfill this criterion. For example, the *B*_2u_ open-shell singlet and the *M*_S_ = 1 component of the *B*_2u_ triplet are degenerate in the MFH model
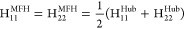
25as the cross-determinant part of the expectation value vanishes. However, the *M*_S_ = 1 component of the triplet
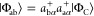
26gives a different energy
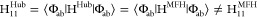
27and so the mean field approximation does not reproduce the correct degeneracy of the components of the triplet. For these reasons, we have preferred to use the full Hubbard model.

Table 1 shows converged energies for a variety of states in units of β, so for fixed electron count a larger value represents a more stable state. At *U* = 0 all 38π-electron states that we have defined are degenerate. Furthermore, the destabilizing effect of a negative Hubbard parameter is clear from the table; for constant *U*, the amount of destabilization rises with the number of π-electrons.

**Table 1:** Hubbard SCF Calculations for Electronic States of the Clar Goblet (*U* = −1.3β)^a^

*N*_e_	state	*E*^tot^/β	ring current			
			*A*	*B*	*C*	*D*
36	(^1^*A*_g_, |Φ_C_⟩) RHF (SALC)	43.12409676997	0.7064	0.6277	0.7276	0.2116
38	(^1^*A*_g_, ) RHF (SALC)	41.90270088783	0.7658	0.6481	0.8025	0.2121
	(^1^*A*_g_, ) UHF (loc)	42.03882640307	0.8144	0.6559	0.8663	0.2151
	(^1^*A*_g_, ) RHF (SALC)	41.90270088783	0.7658	0.6481	0.8025	0.2121
	(^3^*B*_2u_, |Φ_*ab*_⟩) UHF (SALC)	42.03168231735	0.8229	0.6488	0,8837	0.2088
	(^3^*B*_2u_, |Φ_1_⟩) RHF (SALC)	42.00035543807	0.8284	0.6460	0.8800	0.2126
	(^1^*B*_2u_, |Φ_2_⟩) RHF (SALC)	41.81118679423	0.6991	0.6198	0.7173	0.2123
	(^1^*B*_2u_, |Φ_2_⟩) RHF (loc)	42.00035543807	0.8284	0.6460	0.8800	0.2126
	(^1^*B*_2u_, |Φ_3_⟩) RHF (SALC)	42.00035543807				
	(^1^*B*_2u_, |Φ_3_⟩) MCSCF (SALC)	42.00035544596				
	(^1^*A*_g_, |Φ_4_⟩) MCSCF (SALC)	41.81118679416				
	(^1^*A*_g_, |Φ_4_⟩) RHF (SALC)	41.81118679423				
	(CSA) RHF (SALC)	41.93438460925	0.7868	0.6515	0.8292	0.2122
40	(^1^*A*_g_, ) RHF (SALC)	40.52409676997	0.7064	0.6277	0.7276	0.2116

a (Ground-state data in bold.) *N*_e_ is the π electron number for the state. Converged total energies, *E*^tot^, are in units of β. SALC indicates calculations using a symmetry basis; loc indicates a calculation resulting in localised (non-symmetry-adapted) MOs. RHF (UHF) indicate restricted (unrestricted) SCF calculations. Ring currents for symmetry-distinct rings (*A*–*D* in **V** in Figure 1) are calculated using the Hubbard–London method with the same *U* value, and expressed as ratios to the benzene standard. For comparison, the 38 π calculation (RHF or UHF) with *U* = 0 to mimic a pure Hückel calculation gives a repulsion-free total π energy of 54.25270088783 β, and CSA ring currents for *A*, *B*, *C*, *D* of 0.7064, 0.6277, 0.7276, 0.2116, in benzene units.

Our main interest here is in the neutral 38 π-electron states, and we first describe results of the RHF calculations. The degeneracy of the ^1^*A*_g_ closed-shell singlets, , and  follows from the degeneracy of 6*b*_3g_ and 6*b*_1u_ MOs. The ^3^*B*_2u_ open-shell triplet exhibits the most stable of the converged RHF energies, in line with the expression for H_11_^Hub^given in eq 21. The ^1^*B*_2u_ open-shell singlet, on the other hand, has a RHF(SALC) converged energy that is less stable than the triplet, by 2*U*tr(**r**^*a*^**r**^*b*^). However, the same calculation where the SALC basis set is not used, converges to give MOs where *a* and *b* are localized almost completely on the top and the bottom halves of the molecules, respectively. The consequence is that tr **r**^*a*^**r**^*b*^ ≈ 0, resulting in effective degeneracy with the *B*_2u_ triplet. The physical interpretation is that localization reduces electron–electron interaction and enhances stability.

The SALC RHF converged energy for |Φ_3_⟩ is also degenerate with the triplet, as symmetry implies that **r**^*a*^ = **r**^*b*^ (c.f. eq 21). The SALC SCF calculation for |Φ_4_⟩ gives the least stable result. We observe that |Φ_3_⟩, |Φ_4_⟩ are multideterminantal, and therefore we can conduct an MCSCF procedure for the CI function
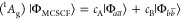
by simultaneous orbital and CI optimizations, giving rise to two converged states. Degeneracy of  and  implies that the first CI iteration gives Φ_3_ and Φ_4_ as initial multiconfigurational states. The CI interaction matrix element *H*_34_ ensures that one MCSCF state is more stable than ^3^*B*_2u_ on convergence, and the other is less stable than ^1^*B*_2u_. The splitting, however, is in the ninth decimal place, smaller by orders of magnitude than the 23 meV (0.0085β) indicated by Mishra et al.^1^ The ordering of states so far is thus in accord with Ovchinnikov’s rule, but only by a whisker, and is revealed only by calculations carried out with high precision.

We now discuss the UHF states, and immediately observe that a symmetry unconstrained calculation on  produces the most stable state of all, where orbitals *a* and *b* have become localized. The degenerate 6*b*_3g_ and 6*b*_1u_ become nondegenerate in the Hubbard field with a splitting of 0.271β = 0.73 eV. This UHF state has *M*_S_ = 0, but is not a pure singlet. Furthermore, the MOs do not transform as irreducible representations of *D*_2*h*_, although overall charge distributions follow the molecular symmetry. The energy expression for the UHF CSF is
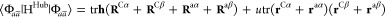
28

The localization of *a* and *b* to separate halves of the molecule ensures that tr **r**^aα^**r**^aβ^ = 0, and explains the stability of the state. We have not been able to obtain an equivalent localized UHF state for the  case.

The *M*_S_ = 1 state, |Φ_*ab*_⟩, is also not a strict triplet in the UHF approximation. We obtained the low energy result in the table with a SALC basis set. It should be noted, however, that the *a* and *b* orbitals belong to the α spin shell and the wave function is invariant to a unitary transformation among this set. The delocalized *a* and *b* MOs we have obtained, therefore, can be rotated into localized form without affecting the energy and the stationary properties of the wave function.

The UHF singlet in our calculations lies 0.007β = −19.3 meV lower than the UHF triplet, and this reproduces the findings of Mishra et al.^1^ It has been stated that there are apparently no exceptions to the Ovchinnikov rule,^7,60,61^ and it seems that the Clar goblet is not an exception. Interestingly, at one stage Clar himself put forward a speculative model by which **V** could attain stability though Dewar-style “para” bonds (see structure XXXIII in ref (24)).

For energy functionals derived from kets , , Φ_*ab*_, Φ_1_, Φ_2_, and , we can conduct Hubbard–London finite field SCF calculations using the McWeenyan many-shell SCF method.^58^ For Φ_3_ or Φ_4_, more general MCSCF-type solution techniques are required, as the CI interaction matrix element for complex MOs cannot be written in terms of densities alone.

At this stage we have a favored candidate for the ground state. What currents are to be expected for this state? Table 1 also gives Hubbard–London results for the symmetry-distinct ring currents (rings *A* to *D* in structure **V** of Figure 1). There are only minor differences between Hubbard–London maps for different states. Rings *A* and *C* host the largest currents, and *D* the smallest, with the ordering of *A* and *C* depending on the state. Overall, however, the picture is very much the same as was found with the Hückel–London model. Both models give the same broad island-isthmus pattern of diatropic current for all states considered. Figure 3 would describe the Hubbard–London results for all states, with only minor modifications.

Finally, we compare the Hückel– and Hubbard–London results with current-density maps from methods at higher levels of theory. For this we use ab initio and Pseudo-π calculations.

**Figure 4:**
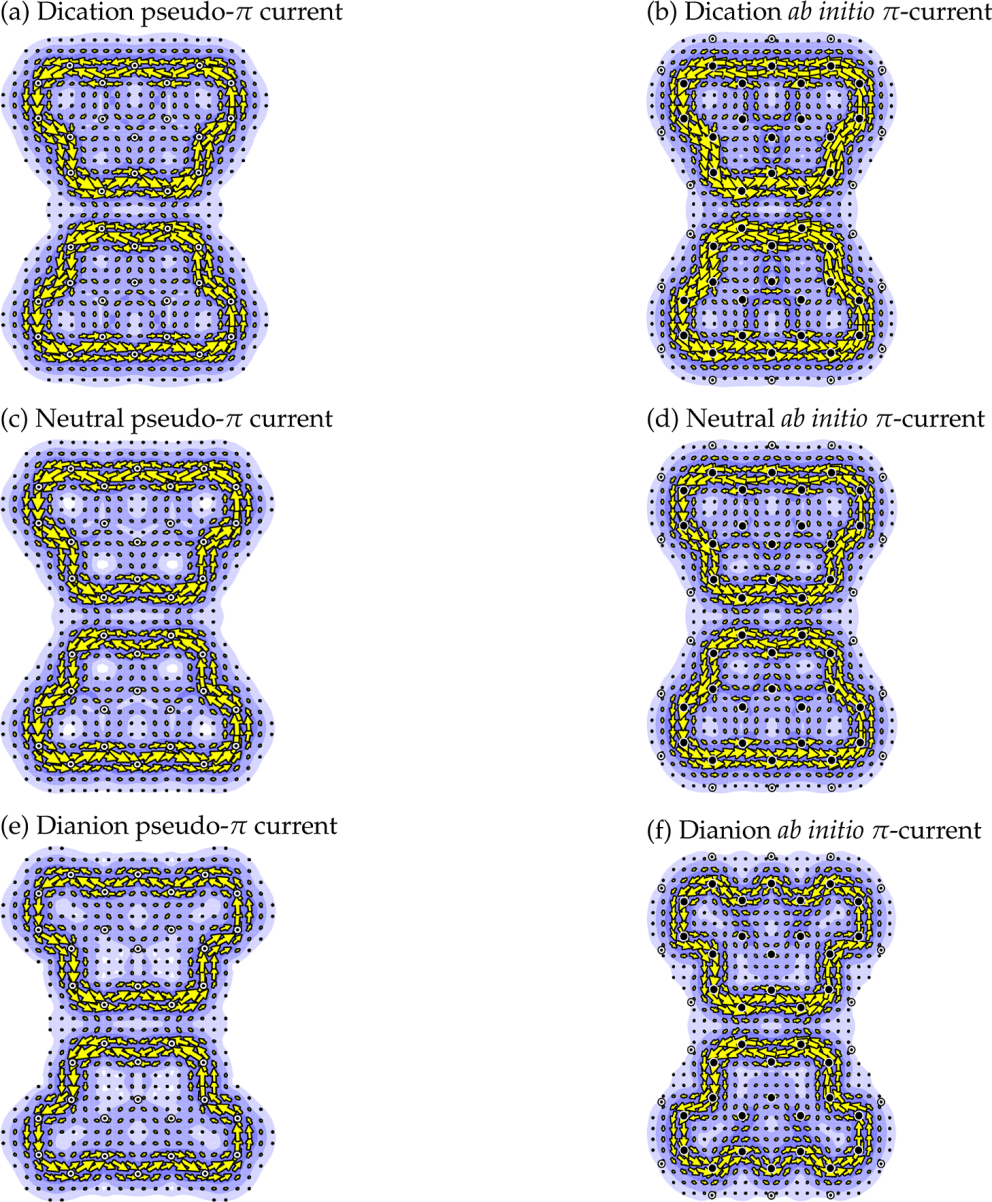
Ipsocentric calculations of π-current induced in the Clar goblet for 36π, 38π, and 40π charge states. The first column shows pseudo-π maps for cation, neutral, and anion; in this simulation of π-current with a σ-basis the plotting plane is the molecular plane. The second column shows the π-current for the same systems derived by summing orbital contributions from all-electron calculations plotted at a height of 1 au above the molecular plane. Arrows represent current per unit magnetic field projected onto the plotting plane; shading represents the modulus of current per unit magnetic field. Filled circles mark positions of C centers, and dot-filled circles the positions of H and pseudo-C centers, all projected into the plotting plane.

Maps for dication, neutral and dianion, obtained with both methods all used these fixed geometries, for which the closed-shell Hartree–Fock procedure generates a ^1^*A*_g_ state in all cases. Figure 4 reports both sets of maps. Ab initio maps (Figure 4b,d,f) for all three charge states are broadly consistent with the Hückel–London and Hubbard–London models, showing well separated circulations on the two wings of the molecular bow-tie, both diatropic in sense, and concentrated on the carbon perimeter. Maps of σ-current in the same plotting plane (not shown here) exhibit the usual paramagnetic vortices at ring centers and an exterior diatropic circulation around the H perimeter, characteristic of a localized σ-bond framework.^62^ The pseudo-π maps (Figure 4a,c,e) are consistent with the full ab initio maps, giving the same pattern of twin diatropic perimeter circulations, relatively minor variation with total charge, and similar strength to the benzene π ring current. The maximum value of the induced current per unit field in the plotting plane, *j*_max_, is 0.080 for benzene, and 0.087, 0.073, and 0.069 for **V**^2+^, **V**^0^ and **V**^2–^ respectively at the pseudo-π level. The ab initio π maps have *j*_max_ values of 0.078 for benzene, and 0.093, 0.070, and 0.073 for **V**^2+^, **V**^0^ and **V**^2–^ respectively. Again, the overall pattern of π-current is remarkably consistent across methods, charge, and states.

In ipsocentric methods, total π maps of induced current density can be partitioned into additive occupied-orbital contributions that individually obey the selection rules described earlier. The pattern of perimeter current and relative inactivity in the interior of each wing is similar to that found in large nanographenes, as mentioned earlier. A similar analysis in terms of constructive and destructive interference of contributions^57^ applies here. Four canonical molecular orbitals (CMOs), each at the top of its symmetry stack in the 36π cation, and also occupied in the 38π and 40π systems, account for the majority of current in the total π maps for the dication. The contributions of the four CMOs remain important for the neutral and dianion maps, but a further orbital contributes significantly on each addition of two π electrons to the total count. For full detail, contributions from more orbitals lower down the stacks are needed. Interestingly, the CMOs corresponding to the nonbonding shell contribute only weakly when occupied. The balance of localized and delocalized effects on currents in medium-sized benzenoids would repay further study.

## 4 Conclusions

Accounting for the ground state of the Clar goblet in terms of qualitative models is clearly a delicate matter, but the prediction of its ring current map proves to be more robust. To a first approximation, at all levels the map for the neutral is essentially the average of the closed-shell maps for the dication and the dianion. All indications from the calculations presented here are that the Clar goblet supports a diatropic/aromatic perimeter π-current on each wing of the molecular bow-tie, with only minor leakage across the central ring.

Indications from calculations at other levels of theory support this claim for insensitivity of the maps. For example, an ACID plot for the singlet open-shell Clar goblet at the π-UB3LYP/6-31G(d,p) level^9^ is consistent with localization of diatropic current on the wing perimeters and minimal communication across the isthmus. The sharp separation of properties of the central hexagonal ring and rings in the wings is also evident in various measures of aromaticity obtained from all-electron and π-only UB3LYP and CASSCF(2,2) calculations, as reported in the same reference. In general, diatropic currents seem not to be sensitive to moving from the CHF to the DFT levels of theory.^63^

**Figure 5:**
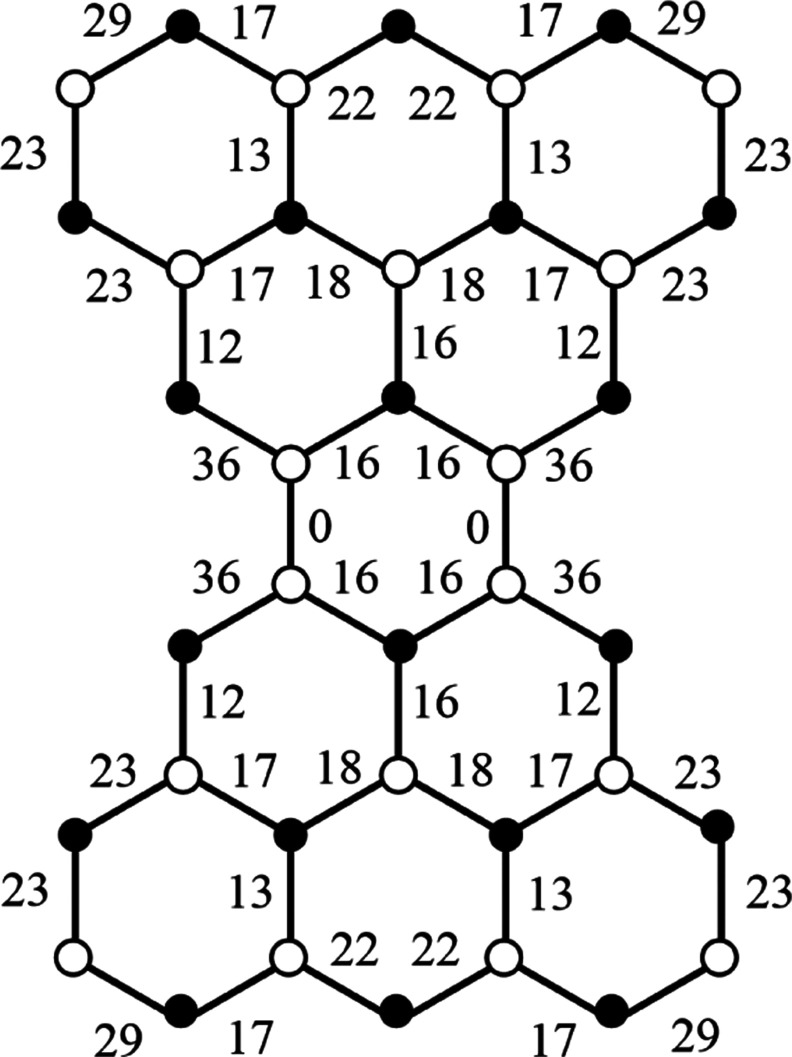
Pauling bond orders in the Clar goblet. The 2704 = 52 × 52 possible maximum matchings represent resonance structures with 18 double bonds and two unpaired electrons [on nonadjacent sites (filled circles), one in each wing]. The integer *q* associated with an edge implies that it carries a double bond in 52*q* matchings, and hence has Pauling bond order 0 ≤ (*q*/52) < 1. Bond orders sum to 1 at each spin-free site (open circle), as these sites participate in exactly one formal double bond in every maximum matching.

In fact, pseudo-π maps (not shown here) for charge states +1, 0, −1 of the trapezoidal five-ring benzenoid C_19_H_11_ corresponding to half of the goblet. Are essentially identical to the maps for one lobe of the current in the goblet itself. This is as expected from the magnetic passivity of the central hexagonal ring of **V**, and from the easy graph-theoretical observation that the bridging bonds in that hexagon are formally single in all diradical resonance structures of **V**. (See Figure 5 for the pattern of bond orders in the neutral Clar goblet, where a Pauling bond order for each edge is defined by the number of times the edge appears as a double bond in a maximum matching expressed as a fraction of the total number of maximum matchings). A similar argument, based on conventional Pauling Bond Order, has been advanced to rationalize the separation of the π-map of closed-shell perylene into naphthalene-like islands^64^ (but see ref (65)).

For practical purposes, the Hückel–London approach with the adaptation for open shells described here gives a qualitatively correct picture of the ring currents for this iconic system. Calculations of this type are easy to carry out and require no more than the molecular graph, and an estimate of either atomic coordinates or ring areas.^25,48^

## Notes

After submission of the present paper, a new preprint^66^ reported synthesis of a sterically protected Clar goblet, in macroscopic amounts, with characterisation of magnetic properties.
